# Toxic Effects of *Rhamnus alaternus*: A Rare Case Report

**DOI:** 10.1155/2015/182951

**Published:** 2015-07-01

**Authors:** H. Ben Ghezala, N. Chaouali, I. Gana, S. Snouda, A. Nouioui, I. Belwaer, J. Ouali, M. Kaddour, W. Masri, D. Ben Salah, D. Amira, H. Ghorbal, A. Hedhili

**Affiliations:** ^1^Teaching Department of Emergency and Intensive Care Medicine, Regional Hospital of Zaghouan, Street of Republic, 1100 Zaghouan, Tunisia; ^2^Research Laboratory of Toxicology-Environment LR12SP07, Laboratory of Toxicology, Center for Emergency Medical Assistance, Montfleury, 1008 Tunis, Tunisia

## Abstract

In Tunisia, there are about 478 species of plants commonly used in folk medicine. Medicinal plants and herbal remedies used are responsible for 2% of intoxications listed by Tunisian National Poison Center. Most cases are related to confusion between edible plants and toxic plants lookalikes or to an excessive consumption of therapeutic plants. We report the case of a 58-year-old man admitted to the Emergency Department of the Regional Hospital of Zaghouan (Tunisia), with renal failure and rhabdomyolysis. The patient reported having daily consumption of a homemade tea based on *Mediterranean Buckthorn* roots, during the last 6 months to treat type 2 diabetes. The aim of this work was to establish an association between the consumption of the herbal remedy and the occurrence of both renal failure and rhabdomyolysis. No similar cases have been reported in recent literature.

## 1. Introduction

Herbal remedies have been used for centuries to treat a variety of diseases.* Mediterranean Buckthorn (Rhamnus alaternus*) has been used for therapeutic purposes and no toxicity effects have been documented.* Rhamnus alaternus* (Rhamnaceae) is a small tree located mainly in the north of Tunisia, where it is known as “Oud El-Khir.” It has traditionally been used as a diuretic, laxative, hypotensive drug and for the treatment of diabetes, hepatic, and dermatologic complications [1, 2]. Previous phytochemical studies have shown potent antioxidant, free radical scavenging, antimutagenic and antigenotoxic activities of flavonoids and phenol isolated from* Rhamnus alaternus* roots and leaves [3, 4].

## 2. Case Report

On February 1, 2013, a 58-year-old man was admitted to the Emergency Department of the Regional Hospital of Zaghouan (Tunisia), with dizziness, weakness, anorexia, and dyspnea. His blood pressure was 130/60 mmHg. The patient has a 15-pack-year history of smoking. He was a mason by occupation. He had 20-year back history of pulmonary tuberculosis and type 2 diabetes revealed one year ago. Two days before his admission, the patient experienced nausea, vomiting, anuria, and hematuria. He reported having daily consumption of a homemade drink based on* Rhamnus alaternus* roots, during the last 6 months, to control his blood glucose levels. On physical examination, the patient had myalgia. He had no other clinical signs.

Cytological reports and sputum smear were negative (three times) for pulmonary tuberculosis. Hepatitis B and hepatitis C serology were also negative. Chest X-ray was normal; blood and urine culture were negative. In renal ultrasonography, there was a significant difference in kidney sizes and the corticomedullary differentiation was altered. Laboratory tests showed glucose 14.44 mmol/L, creatinine 1190 *μ*mol/L, blood urea nitrogen 66.77 mmol/L, creatine phosphokinase (CPK) 2129 UI/L, pH 7.10, a CRP of 8.7 mg/L, and a normal coagulation profile (Table 1). Three dialysis sessions were performed.

### 2.1. Toxicological Analyses

Samples of the herbal decoction were obtained from the patient's wife. It was a dark brown suspension with fine brown deposit and a clear supernatant. It smelled a strong penetrating odor. Samples of both* Rhamnus alaternus* root and its decoction were sent to be analyzed in the Laboratory of Toxicology in the Center for Emergency Medical Assistance of Tunis in Tunisia.

### 2.2. Extraction Procedures

After the authenticity and the botanical identification of the species were confirmed according to the “Flore de la Tunisie” [6] phytochemical compounds were extracted from the medicinal decoction using routine methods including liquid-liquid extraction procedures with further analysis by gas chromatography/mass spectrometry (GC-MS). The solvents used were dichloromethane, ethyl acetate, and chloroform at different pH values (1.0, 7.0, and 9.0). The different extracts were dehydrated over anhydrous sulfate. The dry residue was diluted with 2 milliliters of ethyl acetate. One or 2 *μ*L was analyzed by GC-MS. Dried roots of “*Rhamnus alaternus*” were reduced to small fragments and macerated in a water-methanol mixture (1 : 2) during 4 h with magnetic stirring. 24 hours later, the extract was filtered and the alcoholic layer was evaporated. The aqueous layer was collected in a separating funnel and had been alkalinized by the addition of ammonia (NH_4_OH) and then extracted with dichloromethane by liquid-liquid extraction procedures. The organic phase was dehydrated over anhydrous sulfate and concentrated to 1 mL and then analyzed by GC-MS.

### 2.3. Chromatographic Conditions

The gas chromatograph-mass spectrometer used was a Hewlett Packard 5890-II (Agilent Technologies) fitted with a manual injector and HP5-MS (0.25-*μ*m) capillary column (30 m long and 0.25 mm i.d.). The injection volume was 2 *μ*L; the compounds were separated with helium (carrier gas) at a flow rate of 1 mL/min. The operating conditions were as follows: the injector was programmed to 250°C at 10°C·s^−1^ and held for 2 min. The oven was programmed from 50°C (2 min) to 100°C at 25°C·min^−1^ and then to 200°C at 10°C·min^−1^ (2 min). MS detection was achieved in scan mode for qualitative analysis. Run time was 16 min.

## 3. Results

**Figure 1:**
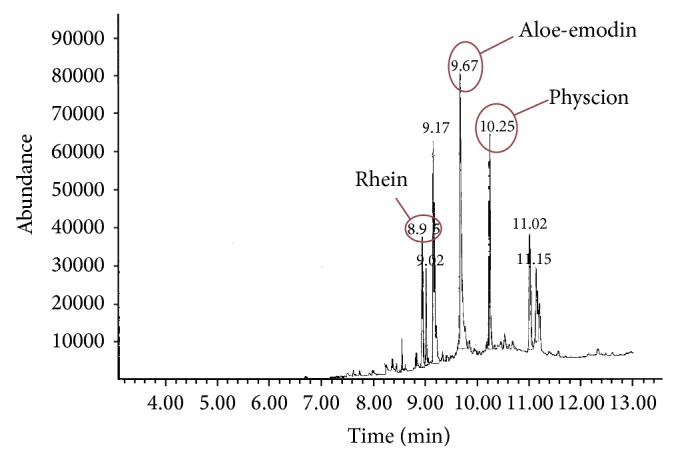
Original chromatogram of herbal tea extract (scan mode).

Screening by GC-MS of both* Rhamnus alaternus* roots and infusion extracts revealed the presence of anthraquinone glycosides such as 4,5-dihydroxy-9,10-dioxoanthracène-2-carboxylic acid (rhein), 1,8-dihydroxy-3-(hydroxymethyl)-9,10-anthracenedione (aloe-emodin), and 1,8-dihydroxy-3-methoxy-6-methylanthracene-9,10-dione (physcion). The retention times were 8.95, 9.67, and 10.25 min, respectively (Figure 1).

Anthraquinone glycosides were detected in a dichloromethane extract and ethyl acetate extract at pH value = 9 and only in a dichloromethane extract at pH value = 7 by GC-MS analysis (Table 2).

## 4. Discussion

Blood chemistry tests performed before dialysis revealed renal nitrogen retention (serum creatinine of 1190 *μ*mol/L and blood urea nitrogen of 66 mmol/L). Moreover, normochromic and normocytic anemia, hypocalcemia, and the loss of corticomedullary differentiation observed in our patient suggest a chronic renal insufficiency. From a biochemical point of view, major hyperglycemia (14.44 mmol/L) could be one of the underlying factors, which lead to the diagnosis of diabetic nephropathy. According to authors, this nephropathy was most likely aggravated by the potential toxic effect of anthraquinone glycosides found in* Rhamnus alaternus* infusion extract. Anthraquinones are a group of functionally diverse chemicals structurally related to anthracene, known to be present in the roots and bark of numerous plants of the genus* Rhamnus* such as senna, cascara, aloe, frangula, and rhubarb used for their laxative properties [7].

**Figure 2:**
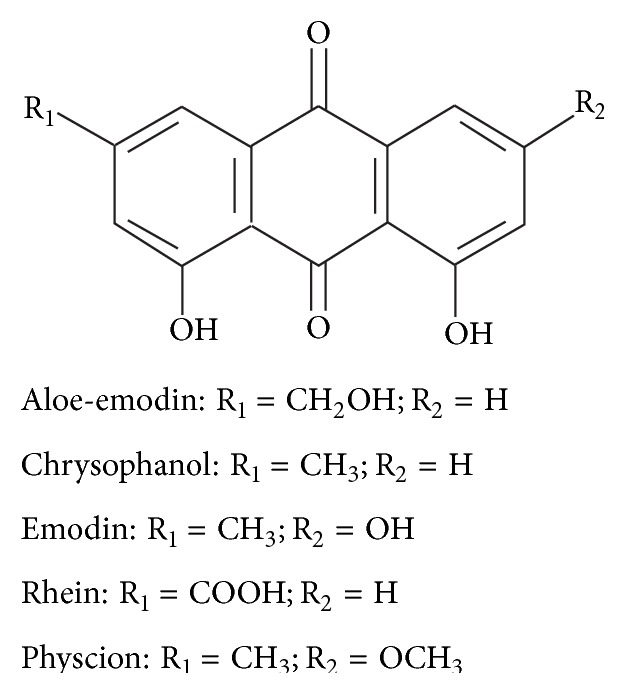
Chemical structure of anthraquinone glycosides [5].

Anthraquinone glycosides including emodin, physcion, aloe-emodin, rhein, and chrysophanol are nowadays well recognized as important biologically active components (Figure 2) [8]. Recently, it was reported that anthraquinones exert a wide range of biological activities including antifungal, antimicrobial, and anticancer properties other than the well-known laxative action on the gastrointestinal apparatus [9–11]. Besides health benefits of anthraquinones, it was also reported that they have a cell toxicity effect. In fact, a study exploring anthraquinones toxicity on Sprague Dawley (S.D.) rats showed that the oral administration of these compounds for 13 weeks induced nephrotoxicity as renal tubule epithelial cells swelled and denatured in tissue slice examination. Anthraquinones were responsible for the activation of mitogen activator protein kinase (MAPK), which causes the arrest of cellular cycle, inhibits epithelial cells proliferation, and contributes to nephrotoxicity [12].

Moreover, investigations revealed increased levels of serum CPK 2129 UI/L, hyperkaliemia of 4.88 mmol/L, and widespread muscle pain on the physical examination, which constitute the diagnostic hallmark of rhabdomyolysis [13]. In the present case, rhabdomyolysis did not have an obvious explanation; there was no ischemia, trauma, or drug intake that could explain it. Nevertheless, we know that one of the rare causes of rhabdomyolysis is metabolic disorders; rhabdomyolysis has been described in chronic hypophosphatemia and hyponatremia, and the most common cause is chronic hypokalemia as it can be seen during treatment with diuretics, in hyperemesis gravidarum or during acute diarrhea episodes [14–17].

In the reported case, the patient has experienced an episode of acute diarrhea one week before his admission to the hospital; rhabdomyolysis could be related to a severe hypokalemia that results from the rapid loss of extracellular potassium losses via gastrointestinal route. In fact anthraquinone glycosides have a strong potential to deplete potassium by stimulating the intestinal secretion of water and electrolytes (K^+^, Na^+^, Cl^−^, etc.) [18], and these glycosides are poorly absorbed from the gastrointestinal tract but are cleaved by gut bacteria to produce aglycones that are more readily absorbed and are responsible for the purgative properties of herb-based stimulant preparations [19].

Long-term therapy with anthraquinone glycosides can alter the body's normal balance of fluids and minerals, which can cause dehydration, severe hypokalemia hyponatremia, asthenia, and anorexia [20, 21]. In addition, lysis of muscle cells releases toxic intracellular components in the systemic circulation that leads to electrolyte disturbances, hypovolemia, metabolic acidosis, and acute renal failure [22, 23]. The patient experienced the same symptoms when he was admitted to hospital except hypokalemia, and the patient has hyperkaliemia because of rhabdomyolysis and the release of intracellular potassium into the plasma. Furthermore, the destruction of muscle cells results in the creation of a “third space” where substantial amounts of water and Na^+^ are concentrated [24, 25], causing hypovolemia and acute renal failure. Organic and phosphoric acids released from the muscle cell lead to metabolic acidosis and increase the anion gap due to the overproduction of organic acids [26].

In summary, the patient who refused to take any medication to control his blood glucose levels except herbal medication has an undiagnosed diabetic nephropathy aggravated by acute renal dysfunction and rhabdomyolysis. All metabolic disorders are mainly imputed to the toxic effects of the anthraquinone glycosides. We noticed that patient completely recovered and symptoms regressed completely when stopping herbal infusion ingestion. Blood chemistry tests performed after dialysis were all normal.

## 5. Conclusion 

*Rhamnus alaternus* can be toxic when used in an abusive way beside its strong antibacterial, antioxidant, and antidiabetic activities. To our knowledge, this is the first report of a case of renal failure and rhabdomyolysis which is possibly associated with a chronic consumption of* Rhamnus alaternus* roots. We present this case to illustrate the role of both clinical and biological investigations in handling cases of herbal poisonings. We aimed also to increase awareness among emergency physicians about patients presenting to the Emergency Department with unexplained symptoms (renal failure, rhabdomyolysis, etc.) requiring prompt diagnosis so that such life-threatening complications can be avoided.

## Figures and Tables

**Table 1:** Biochemical, hematologic, and blood gas parameters, before and after dialysis.

Blood tests	Before dialysis	After dialysis			Normal ranges (adult male)
		Day 4	Day 7	Day 9	
Glucose	14.44	4.74	4.50	4.55	3.9–6.1 mmol/L
Urea nitrogen	66.77	46.04	39	50.99	2.5–7.5 mmol/L
Creatinine	1190	756	853	811	60–110 *μ*mol/L
Sodium	122	141	120.5	128.1	135–145 mmol/L
Potassium	4.88	2.44	3.61	3.81	3.5–4.5 mmol/L
Calcium	1.4	1.5	0.92	1.19	2.20–2.55 mmol/L

CPK	2129	2163	3399	1230	<195 UI/L
CPK MB	484.1	481.5	154.8	119	0–24 UI/L
Hemoglobin	8.7	8.2	7.4	6.3	12.3–15.3 g/dL
WBC	3.1	2.86	1.68	1.52	4–10 × 10^3^/mm^3^
Platelets	382.0	383.0	249.0	262.0	150.0–450.0 × 10^3^/mm^3^

pH	7.10	7.29	7.32	7.38	7.38–7.42
HCO^3−^	5.1	18.2	19.2	18.2	22–26 mmol/L
Anion gap	35.8	25.2	7.21	20.31	16–18 mmol/L
PaO_2_	88	91	88	86	95–98 mm Hg
PaCO_2_	16.2	26	27	28	40–45 mm Hg

**Table 2:** Qualitative screening by gas chromatography-mass spectrometry (GC-MS).

	Dichloromethane extract			Ethyl acetate extract			Chloroform extract		
	pH = 1	pH = 7	pH = 9	pH = 1	pH = 7	pH = 9	pH = 1	pH = 7	pH = 9
Rhein	ND	+	+	ND	ND	+	ND	ND	ND
Physcion	ND	+	+	ND	ND	+	ND	ND	ND
Aloe-emodin	ND	+	+	ND	ND	+	ND	ND	ND

+: detected; ND: not detected.
